# Attributable risk from distributed lag models

**DOI:** 10.1186/1471-2288-14-55

**Published:** 2014-04-23

**Authors:** Antonio Gasparrini, Michela Leone

**Affiliations:** 1 Department Medical Statistics, London School of Hygiene and Tropical Medicine, Keppel Street, WC1E 7HT, London, UK; 2 Department of Epidemiology, Lazio Regional Health Service, Via di Santa Costanza 53, 00198 Rome, Italy

**Keywords:** Attributable risk, Attributable fraction, Distributed lag models

## Abstract

**Background:**

Measures of attributable risk are an integral part of epidemiological analyses, particularly when aimed at the planning and evaluation of public health interventions. However, the current definition of such measures does not consider any temporal relationships between exposure and risk. In this contribution, we propose extended definitions of attributable risk within the framework of distributed lag non-linear models, an approach recently proposed for modelling delayed associations in either linear or non-linear exposure-response associations.

**Methods:**

We classify versions of attributable number and fraction expressed using either a forward or backward perspective. The former specifies the future burden due to a given exposure event, while the latter summarizes the current burden due to the set of exposure events experienced in the past. In addition, we illustrate how the components related to sub-ranges of the exposure can be separated.

**Results:**

We apply these methods for estimating the mortality risk attributable to outdoor temperature in two cities, London and Rome, using time series data for the periods 1993–2006 and 1992–2010, respectively. The analysis provides estimates of the overall mortality burden attributable to temperature, and then computes the components attributable to cold and heat and then mild and extreme temperatures.

**Conclusions:**

These extended definitions of attributable risk account for the additional temporal dimension which characterizes exposure-response associations, providing more appropriate attributable measures in the presence of dependencies characterized by potentially complex temporal patterns.

## Background

Epidemiological studies usually rely on effect summaries based on *ratio measures*, such as relative risk, odds ratio or rate ratio, with the choice depending on the specific study design [1]. Although these measures are ideal for summarizing the association of interest, they offer limited information on the actual impact of the exposure. This information is critical for the planning and evaluation of public health interventions, and it is better provided by *relative excess measures* such as the attributable fraction (AF), or *absolute excess measures* such as the attributable number (AN). Steenland and Armstrong offer a thorough overview on the topic [2]. Here we generally refer to these summaries as *attributable risk measures*.

Problems in the definition of these measures may arise in the presence of *delayed associations*, occurring when an exposure generates a risk lasting well beyond the exposure period. Researchers in different fields, during the last thirty years, have proposed approaches for modelling this type of association [3-6]. In time series analysis, a popular approach is based on distributed lag models (DLMs) [7,8], generalized to distributed lag non-linear models (DLNMs) when including non-linear exposure-response associations [9,10]. Recently, the DLNM framework has been extended beyond time series data for modelling such dependencies, defined *exposure-lag-response associations*, in different study designs [11]. However, attributable risk measures have not been developed for the DLM and DLNM class, with the result that their current definitions do not take into account the additional temporal structure in the exposure-response association. Previous work investigated the issue in time series analysis, although without producing a general approach [12,13].

In this contribution, we extend this research and attempt to formalize the definition of attributable risk measures within the DLNM modelling framework. In particular, we illustrate how complex non-linear and temporal patterns can be accounted for in the computation of the attributable risk. Also, we show how attributable components related to different exposure ranges can be separated. We propose an example on the estimation of the health burden attributable to temperature with time series data, a problem which has received quite a lot of interest recently due to climate change predictions. However, the approach can be easily applied to other exposure-lag-response associations. The method is implemented in simple functions developed within the R software package and provided as additional files.

## Methods

### Attributable risk measures

A general definition of the attributable fraction AF_*x*_ and number AN_*x*_ for a given exposure *x* can be provided by:

(1a) $$\begin{array}{ll} {\text{AF}}_{x} & =1-\exp\left(-\beta_{x}\right)\;,\; \end{array}$$

(1b) $$\begin{array}{ll} {\text{AN}}_{x} & =n\cdot {\text{AF}}_{x}\;,\; \end{array}$$

with *n* as the total number of cases. The parameter *β*_*x*_ used in Eq. (1a) represents the risk associated with the exposure, and it usually corresponds to the logarithm of a ratio measure such as relative risk, relative rate or odds ratio. It is generally obtained from regression models while adjusting for potential confounders. The general definition of *β*_*x*_ used here refers to the association with a specific exposure intensity *x* compared to a reference value *x*_0_. For linear exposure-response relationships, the association can also be reported as *β*·*x*, where in this case *β* refer to a unit increase in *x*. For binary variables reporting presence/absence of the exposure, Eq. (1a) simplifies to AF=(RR−1)/RR, with RR as relative risk, as reported by Steenland and Armstrong [2]. We keep the more general definition of *β*_*x*_, which is easily applicable to non-linear exposure-response relationships, throughout the manuscript.

The theoretical nature of these effect measures is based on a *counterfactual*, where the observed condition is compared with a reference state which never occurred. This state postulates that the same population is followed in an identical situation where only the exposure level changes to the reference value *x*_0_. Typically, such a reference is represented by the absence of association, meaning *x*_0_=0 and $\beta_{x_{0}}=0$. However, different counterfactual conditions can be used, for example a lower exposure which can be determined by an intervention. In this case the quantity *β*_*x*_ can be simply re-parameterized as $\beta_{x}^{*}=\beta_{x}-\beta_{x_{0}}$, and Eq. (1) still applies.

Eq. (1a) can be extended to define the risk attributable to multiple exposures *x*_1_,…,*x*_*p*_:

(2) $${\text{AF}}_{x_{1},\ldots ,x_{p}}=1-\exp\left(-\overset{p}{\underset{i=1}{\sum }}\beta_{x_{i}}\right)=1-\overset{p}{\underset{i=1}{\prod }}\left(1-{\text{AF}}_{x_{i}}\right)\;,$$

with ${\text{AN}}_{x_{1},\ldots ,x_{p}}$ obtained by substituting Eq. (2) in Eq. (1b) [2]. For the specific form of Eq. (2), it should be noted that ${\text{AF}}_{x_{1},\ldots ,x_{p}}\leq {\text{AF}}_{x1}+\ldots +{\text{AF}}_{\text{xp}}$, *i.e.* the sum of the attributable risk measured for individual exposures is usually higher than their concurrent attributable risk.

### A review of the DLNM modelling framework

The basic idea underpinning the development of DLNMs is that the risk at time *t* can be described as the weighted sum of effects cumulated from a series of exposures $x_{t-\ell_{0}},\ldots ,x_{t-L}$ experienced in the past over the lag period *ℓ*=*ℓ*_0_,…,*L*, with *ℓ*_0_ and *L* corresponding to minimum and maximum lags, respectively. The risk can be described by the function *f*(*x*), determining the exposure-response, and the function *w*(*ℓ*), specifying the lag-response, related to the weights given to exposures at different lags *ℓ*. These functions are combined in a bi-dimensional *exposure-lag-response functionf* · *w*(*x*,*ℓ*). Algebraically, the risk is defined by a function *s*(*x*,*t*;***η***), written in terms of parameters ***η*** as:

(3) $$\begin{array}{lll} s(x,t;\eta ) & =\overset{L}{\underset{\ell_{0}}{\int }}f\;\cdot \;w(x_{t-\ell },\ell )\;\mathrm{d\ell}\; & \;\\ & \approx \;\overset{L}{\underset{\ell =\ell_{0}}{\sum }}f\;\cdot \;w(x_{t-\ell },\ell )=w_{x,t}^{T}\eta \;\text{.}\; \end{array}$$

The function *s*(*x*,*t*) is computed as the approximate integral of the exposure-lag-response function over the lag dimension, representing the cumulated risk over the lag period. The parameterization in the final step of Eq. (3) is obtained through a *cross-basis*, involving a tensor product between the basis chosen for *f*(*x*) and *w*(*ℓ*), generating the transformed variables **w**_*x*,*t*_ linearly combined with the parameters ***η***. Simpler DLMs are defined by Eq. (3) by assuming *f*(*x*) as linear. Algebraic details and additional information are provided elsewhere [11]. The cross-basis is specified with a reference value *x*_0_ used later as a centering point for the function *f*(*x*), which is used to define the counterfactual condition.

The complex parameterization of exposure-lag-response associations provided by Eq. (3) can be more easily interpreted by computing effect summaries from the original parameters ***η***. Specifically, the bi-dimensional exposure-lag-response risk surface modelled through *f* · *w*(*x*,*ℓ*) can be expressed by a grid of effect summaries *β*_*x*,*ℓ*_, each interpreted as the association with an exposure *x* at lag *ℓ* versus the reference value *x*_0_. For a given time *t*, the cross-basis parameterization in (3) can be re-expressed as:

(4) $$w_{x,t}^{T}\eta =\overset{L}{\underset{\ell =\ell_{0}}{\sum }}\beta_{x_{t-\ell },\ell }\;\text{.}$$

This *overall cumulative* association is composed of the sum of contributions *β*_*x*,*ℓ*_ from exposures $x_{t-\ell_{0}},\ldots ,x_{t-L}$ experienced within the lag period. Algebraic definitions have been previously provided [11].

### Forward and backward perspectives

**Figure 1:**
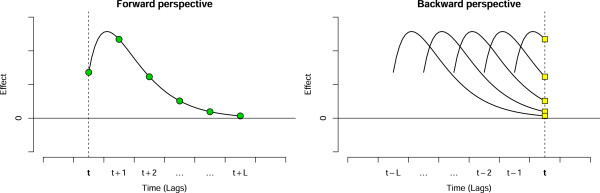
Conceptual model for the interpretation of exposure-lag-response associations: forward (left panel) and backward (right panel) perspectives.

The term *β*_*x*,*ℓ*_ for each intensity *x* can be interpreted using two complementary perspectives, illustrated graphically in Figure 1. From a *forward* standpoint, looking from current exposure to future risks, the terms *β*_*x*,*ℓ*_ are the contributions from the exposure *x*_*t*_ occurring at time *t* to the risk at times *t*+*ℓ*_0_,…,*t*+*L*, identified by green circles. From a *backward* standpoint, looking from current risk to past exposures, the terms *β*_*x*,*ℓ*_ are the contributions to the risk at time *t* from exposures $x_{t-\ell_{0}},\ldots ,x_{t-L}$ experienced at *t*−*ℓ*_0_,…,*t*−*L*, identified by yellow squares. The underlying curve in Figure 1 depicting such associations is called the *lag-response curve* related to a given exposure intensity *x*. The sum of these contributions over the whole lag period can be interpreted as the overall cumulative risk.

### Attributable risk from DLNMs

The effect summaries provided above can be used for defining attributable risk measures within the DLNM framework. The idea is to treat the associations with exposures at different lags as independent contributions to the risk. A neat definition can be developed using a backward perspective, assuming the risk at time *t* as attributable to a series of exposure events in the past. The backward attributable fraction b-AF_*x*,*t*_ and number b-AN_*x*,*t*_ at time *t* are obtained by substituting Eq. (4) in Eq. (2):

(5a) $$\begin{array}{ll} {b-\text{AF}}_{x,t} & =1-\exp\left(-\overset{L}{\underset{\ell =\ell_{0}}{\sum }}\beta_{x_{t-\ell },\ell }\right)\;,\; \end{array}$$

(5b) $$\begin{array}{ll} {b-\text{AN}}_{x,t} & ={b-\text{AF}}_{x,t}\cdot n_{t}\;,\; \end{array}$$

with *n*_*t*_ as the number of cases at time *t*. This structure is consistent with the configuration of the regression model usually applied to fit the data, where the risk at time *t* is associated with lagged exposures at times *t*−*ℓ*. The definition of backward attributable risk requires an extended version of the counterfactual condition accounting for the additional lag dimension: b-AN_*x*,*t*_ and b-AF_*x*,*t*_ are interpreted as the number of cases and the related fraction at time *t* attributable to past exposures to *x* in the period *t*−*ℓ*_0_,…,*t*−*L*, compared to a constant exposure *x*_0_ throughout the same period.

An alternative version can be obtained using a forward perspective. Among other possible definitions, forward attributable number f-AN_*x*,*t*_ and fraction f-AF_*x*,*t*_ can be defined as:

(6a) $$\begin{array}{ll} {f-\text{AF}}_{x,t} & =1-\exp\left(-\overset{L}{\underset{\ell =\ell_{0}}{\sum }}\beta_{x_{t},\ell }\right)\;,\; \end{array}$$

(6b) $$\begin{array}{ll} {f-\text{AN}}_{x,t} & ={f-\text{AF}}_{x,t}\cdot \overset{L}{\underset{\ell =\ell_{0}}{\sum }}\frac{n_{t+\ell }}{L-\ell_{0}+1}\;\text{.}\; \end{array}$$

This alternative version has some advantages if compared to the backward definition. First, the counterfactual condition is simpler: f-AF_*x*,*t*_ and f-AN_*x*,*t*_ are interpreted as the fraction and number of future cases in the period *t*+*ℓ*_0_,…,*t*+*L* attributable to the single exposure *x* occurring at time *t*, compared to *x*_0_. Moreover, the overall cumulative risk $\sum \beta_{x_{t},\ell }$ for a given exposure *x*_*t*_ in (6a) is available also when the bi-dimensional exposure-lag-response is reduced to uni-dimensional exposure-response relationship, a step often needed in multi-site studies [14]. In contrast, all the lag-specific contributions are needed to compute $\sum \beta_{x_{t-\ell },\ell }$ in (5a) for the backward counterpart.

However, the forward version also has an important limitation, related to the fact that the contributions are associated to risks measured at different times. The attributable number f-AN_*x*,*t*_ in (6b) is computed by averaging the total counts experienced in the next *ℓ*_0_,…,*L* times, thus only approximating the lag structure of risks. This approximation is likely to produce some bias, which is expected as an underestimation of the attributable number if compared to the backward version.

### Separating attributable components

The definitions provided in Eq. (5)–(6) can be extended to separate the attributable components related to specific exposures or exposure ranges. This will be used later in the example to single out the contributions from cold and heat in temperature-health associations. Let’s define a range *r*=[*l*,*h*] between low and high exposure limits *l* and *h*. The definition of forward attributable number ${f-\text{AN}}_{x,t}^{r}$ and fraction ${f-\text{AF}}_{x,t}^{r}$ limited to exposures within the range *r* is clear-cut, as they are either equal to the quantities reported in Eq. (6) if *x*∈*r* or zero otherwise. Adopting a backward perspective, a similar definition of ${b-\text{AF}}_{x,t}^{r}$ can be obtained by modifying Eq. (5a) as:

(7) $${b-\text{AF}}_{x,t}^{r}=1-\exp\left(-\overset{L}{\underset{\ell =\ell_{0}}{\sum }}I\left(x_{t-\ell }\in r\right)\beta_{x_{t-\ell },\ell }\right)\;,$$

simply selecting the risk contributions from past exposures included in the range *r*. The related attributable number ${b-\text{AN}}_{x,t}^{r}$ is computed by substituting Eq. (7) into Eq. (5b). Attributable components referring to different ranges can be summed up, as all are defined using the same counterfactual condition of a constant exposure *x*_*ℓ*_=*x*_0_ for the whole lag period *ℓ*=*ℓ*_0_,…,*L*.

The forward version has the additional advantage that for two non-overlapping ranges *r*_1_ and *r*_2_ the sum of the components is equal to the overall attributable risk, namely ${f-\text{AF}}_{x,t}^{r_{1}+r_{2}}={f-\text{AF}}_{x,t}^{r_{1}}+{f-\text{AF}}_{x,t}^{r_{2}}$. In contrast, adopting a backward perspective ${b-\text{AF}}_{x,t}^{r_{1}+r_{2}}\leq {b-\text{AF}}_{x,t}^{r_{1}}+{b-\text{AF}}_{x,t}^{r_{2}}$, as the risks are simultaneously computed for the same time *t* in the like of Eq. (2).

### Total attributable risk

The attributable risk measures provided above can be computed for each of the *i*=1,…,*m* observations in a data set. An estimate of the total attributable number AN_*tot*_ and fraction AF_*tot*_ is provided by:

(8a) $$\begin{array}{ll} {\text{AN}}_{\text{tot}} & =\overset{m}{\underset{i=1}{\sum }}{\text{AN}}_{x,t_{i}}\;,\; \end{array}$$

(8b) $$\begin{array}{ll} {\text{AF}}_{\text{tot}} & ={\text{AN}}_{\text{tot}}/\overset{m}{\underset{i=1}{\sum }}n_{t_{i}}\;\text{.}\; \end{array}$$

The equations above can be applied either to forward or backward attributable risk and to separate components, simply substituting the related attributable numbers in Eq. (8a).

### Computing uncertainty intervals

Analytical formulae for confidence intervals of attributable risk measures are not easily produced [15], and this also applies to the extended versions developed here. Although approximated estimators have been proposed [15,16], in this context the most straightforward approach is to rely on interval estimation obtained empirically through Monte Carlo simulations [17,18]. Basically, we take random samples ***η***^(*j*)^ of the original parameters ***η*** of the cross-basis in Eq. (3) from the assumed multivariate normal distribution with point estimate $\hat{\eta }$ and (co)variance matrix $V(\hat{\eta })$ derived from the regression model. These samples ***η***^(*j*)^ are used to compute $\beta_{x,\ell }^{\left(j\right)}$ for *ℓ*=*ℓ*_0_,…,*L* and each intensity *x*, empirically reconstructing the distributions of the attributable measures defined in Eq. (5)–(8). The related 2.5^th^ and 97.5^th^ percentiles of such distributions are interpreted as 95% empirical confidence intervals (eCI).

## Results

The methods illustrated in the previous section are applied to estimate the all-cause mortality risk attributable to temperature, using daily time series from two cities, London and Rome, in the periods 1993-2006 and 1992-2010 respectively. R scripts and data implementing the method and partly replicating the results are provided as Additional files 1, 2, 3, 4, 5 and 6.

### Modelling strategy

We fitted a standard time series Poisson model allowing for overdispersion, controlling for seasonal and long term trends and day of the week, using a 10 df/year spline and indicator variables, respectively. Model selection is still an issue of current research within the DLNM framework, although simulation studies indicate a good performance of methods based on the Akaike Information criterion (AIC) [11]. Considering the illustrative purpose of the example, we selected a-priori the cross-basis function in Eq. (3) for representing the association between mean daily temperature and mortality, basing our choice on previous analyses. Specifically, the cross-basis is composed of a quadratic B-spline with two equally-spaced knots as the exposure-response function *f*(*x*), and a natural cubic B-spline with three equally-spaced knots in the log-scale as the lag-response function *w*(*ℓ*) over lags 0–25.

In the specific case of temperature where a null exposure condition cannot be defined, a reasonable choice is to center the cross-basis in Eq. (3) to the temperature of minimum risk, as suggested in previous publications [13]. This optimal temperature corresponds to 20°C and 21°C for London and Rome respectively, and it represents the reference point *x*_0_ for the computation of the attributable risk measures. These are obtained for the whole temperature range, and then for cold and heat contributions by separating the associations with temperatures lower or higher than *x*_0_. In addition, the attributable components are separated further in mild and extreme cold and heat by selecting as cut-off values the 1^st^ and 99^th^ percentiles of city-specific distributions, corresponding to 0.4°C and 23.7°C in London and 2.6°C and 28.6°C in Rome.

We derived empirical confidence intervals for backward total attributable numbers and fractions, computed overall and for separated components, by simulating 5,000 samples from the assumed distribution of $\hat{\eta }$.

### Risk attributable to temperature

**Figure 2:**
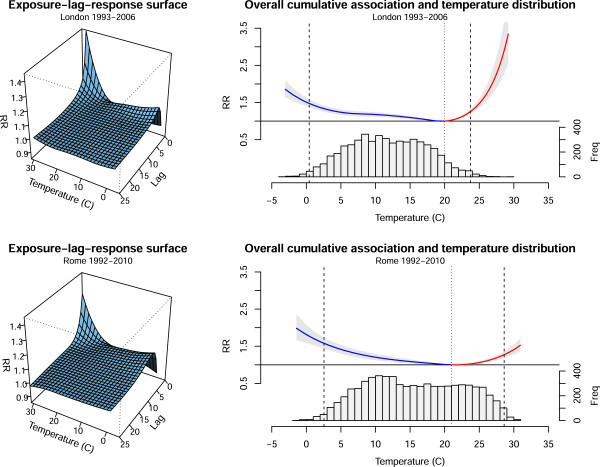
**Association between temperature and all-cause mortality.***Left panels*: 3-D graphs of the exposure-lag-response risk surface. *Right panels*: overall cumulative exposure-response associations with temperature distributions. London 1993–2006 and Rome 1992–2010.

The estimated associations between temperature and all-cause mortality in the two cities are illustrated in Figure 2. The left panels show the bi-dimensional exposure-lag-response surfaces, while the right panels display the overall cumulative exposure-response curves, interpreted as the risk cumulated over the entire lag period of 0–25 days. The associations are represented in the RR scale, with the centering point and the cut-off values for defining extreme cold and heat displayed as dotted and dashed vertical lines respectively.

Table 1 reports the estimates of the total backward and forward attributable fraction b-AF_*tot*_ and f-AF_*tot*_, with 95%eCI. The backward version indicates that overall 13.59% and 12.58% deaths are attributable to temperature in London and Rome within the study periods, respectively. As expected, the corresponding estimates computed forward are affected by a certain degree of negative bias associated to the averaging of future mortality within the lag period, as described above. Nonetheless, the difference is not substantial in this case.

**Table 1:** **Total mortality fraction (%) attributable to temperature, computed backward (*b-AF***_***tot***_**) and forward (*f-AF***_***tot***_**), reported as overall, hot and cold components with 95% empirical confidence intervals (eCI)**

	**Deaths**		**Overall**	**Cold**	**Hot**
London	845,215	b-AF_*tot*_	13.59 (10.04–17.09)	12.95 (9.32–16.38)	0.66 (0.52–0.80)
		f-AF_*tot*_	13.41 (9.72–16.87)	12.84 (9.38–16.33)	0.57 (0.45–0.68)
Rome	395,691	b-AF_*tot*_	12.58 (9.30–15.64)	10.84 (7.37–14.23)	1.74 (1.12–2.37)
		f-AF_*tot*_	12.27 (8.94–15.41)	10.72 (7.19–14.00)	1.55 (0.95–2.13)

London 1993–2006 and Rome 1992–2010.

### Cold, heat and extreme components

The total backward attributable risk is then separated into components due to cold and hot temperatures, defined as those below and above the optimal temperature, respectively. The estimates, computed using Eq. (7), are reported in Table 1. The comparison of the two contributions clearly indicates that cold is responsible for most of the mortality attributable to temperature, with b-AF_*tot*_ equal to 12.95% and 10.84%, compared to 0.66% and 1.74% for heat, in the two cities. Estimates of forward attributable risk are very similar, and as expected their sum is equal to the overall burden, differently than for the backward version.

The analysis is extended by further separating the attributable components into contributions from mild and extreme temperatures, with results summarized in Table 2. For cold temperatures, the contribution from extreme days accounts only for a minimal part of the mortality burden, while still 12.38%–12.48% and 10.27%–10.37% of deaths in London and Rome, respectively, are attributable to mild cold days. This result is expected when inspecting the right panels of Figure 2, with the definition of mild cold including the majority of days in the series for both cities.

**Table 2:** **Total mortality fraction (%) attributable to temperature, computed backward (*b-AF***_***tot***_**) and forward (*f-AF***_***tot***_**), reported as components from mild and extreme hot and cold contributions with 95% empirical confidence intervals (eCI)**

		**Extreme cold**	**Mild cold**	**Mild hot**	**Extreme hot**
London	b-AF_*tot*_	0.55 (0.45–0.64)	12.48 (8.86–15.88)	0.31 (0.23–0.38)	0.36 (0.29–0.43)
	f-AF_*tot*_	0.47 (0.40–0.53)	12.38 (8.98–15.78)	0.29 (0.22–0.35)	0.28 (0.23–0.33)
Rome	b-AF_*tot*_	0.59 (0.47–0.70)	10.37 (6.88–13.63)	1.45 (0.89–2.01)	0.33 (0.25–0.40)
	f-AF_*tot*_	0.47 (0.39–0.54)	10.27 (6.69–13.50)	1.32 (0.75–1.85)	0.25 (0.19–0.30)

London 1993–2006 and Rome 1992–2010.

In contrast, the comparison between the two cities is rather different for the components attributable to mild and extreme hot temperatures. In spite of the stronger risk in London, the attributable fraction is similar for extreme heat and even higher in Rome for mild heat (1.32%–1.45% versus 0.25%–0.33%). This apparent contradiction is explained by the different temperature distribution, and in particular the percentile corresponding to the optimal temperature, corresponding to 93.6^th^ and 72.5^th^ in London and Rome. This result suggests the hypothesis that the population in Rome is more adapted to the range of temperatures corresponding to extreme hot if compared to London, where the population experienced only a few days of unusually high temperatures.

### The harvesting paradox

**Figure 3:**
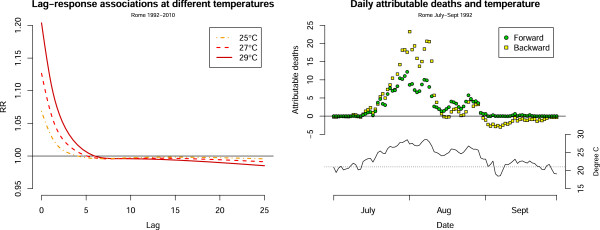
**Lag structure and harvesting paradox.** Left panel: lag-response associations between various temperatures and all-cause mortality. Rome 1992–2010. Right panel: daily number of deaths attributable to heat, computed forward (green circles) and backward (yellow squares), and temperature trend. Rome July-Sept 1992.

Accounting for the additional lag dimension in exposure-lag-response associations involves further complexities in the interpretation of attributable risk measures. We now focus our attention to the association with hot temperature in Rome. The left panel of Figure 3 shows the estimated lag-response curves at various temperatures, computed versus the reference optimal temperatures of 21°C. The graph indicates a strong risk in the first lags, then followed by a protective association at longer lags. This pattern is consistent with the harvesting hypothesis, which assumes that the initial risk is partly discounted by the depletion of the pool of susceptible after an extreme heat event [19]. Also, the extent of harvesting seems more pronounced for extreme temperatures.

This phenomenon has interesting implications. An example is offered by the right panel of Figure 3, illustrating the estimated daily deaths b-AN_*x*,*t*_ and f-AN_*x*,*t*_ attributable to heat, computed backward and forward for the first summer in the time series for Rome, with related temperature trend. As expected, a substantial number of deaths are attributable to temperatures above the optimal value, represented by the horizontal dotted line, in the period mid-July to mid-August. The trend of forward attributable deaths f-AN_*x*,*t*_ closely follows the daily temperatures, consistently with the definition of number of deaths attributable to the temperature in day *t* cumulated in the next *L* days. In contrast, the backward attributable number b-AN_*x*,*t*_ decreases to zero and even becomes negative in late summer days, although the overall cumulative exposure-response in Figure 2 (bottom-right panel) does not show a RR below 1 for any temperature.

This paradox is explained by the counterfactual condition associated with the backward perspective. Specifically, each b-AN_*x*,*t*_ compares the association with the observed temperatures in the past *L* days to a constant exposure *x*_0_. In the presence of harvesting, the observed population becomes ‘healthier’ than the counterfactual population after a series of heat days, due to the depletion of the susceptible pool. This explains the negative attributable numbers for specific combinations of lagged exposures. This fact emphasises that harvesting should not be interpreted as a true protective association at longer lags, but rather as an artefact due to a change in the underlying population following a stress, which affects the counterfactual condition. This issue is relevant when using backward attributable risk measures b-AN_*x*,*t*_ and b-AF_*x*,*t*_ to assess the contribution of specific days. However, similarly to the net overall cumulative risk, the total attributable number b-AN_*tot*_ and fraction b-AN_*tot*_, produced by Eq. (8) and reported in Tables 1 and 2, account for the discount by summing the contributions over the whole series.

## Discussion and conclusions

In this contribution we illustrate an extended definition of attributable risk measures based on the DLNM framework. Consistently with this class of models, such a definition accounts for the complex pattern of potentially non-linear and delayed associations described through exposure-lag-response associations.

Two alternative definitions of attributable risk are proposed, assuming backward or forward perspectives. The former provides more consistent estimators which naturally arise from the structure of the regression model, where distributed lag terms at times *t*−*ℓ* contributes to the risk at time *t*. The forward attributable measures, in contrast, are affected from a negative bias related to the averaging of future counts, which nonetheless is likely to be relatively low. On the other hand, the forward version is well suited for separating the risk in components attributable to different ranges, as their sum matches the overall risk. Furthermore the forward perspective, looking from current exposure to future risk, seems more appropriate for quantifying the health burden due to specific exposure occurrences, as it is based on a more coherent counterfactual condition. Corrections have been proposed in previous works on risk attributable to multiple exposures [20-22], and can be applied to the backward version.

Strictly speaking, the definition given in Eq. 1a is interpreted as the attributable fraction among the sub-population of exposed subjects. In the setting of time series analysis for environmental stressors, the whole population is usually considered as exposed, and this definition can be more generally interpreted as the population attributable fraction. If only a subset is instead exposed, Eq. (5)–(8) can be easily extended using the equations proposed by Steenland and Armstrong [2] for population attributable risk.

Previous papers suggested approaches for producing attributable risk from distributed lag models when applied to heat-mortality associations. Baccini and colleagues applied DLMs and computed attributable risk measures, specifically addressing the issue of harvesting [12]. Honda and colleagues illustrated an analysis on the mortality burden due to heat using DLNMs [13]. However both approaches are limited, as the former assumes a linear threshold form of the exposure-response, while the latter averages the non-linear risk across the whole temperature range. In this paper we offer a formal and more consistent definition of such attributable risk measures.

An advantage of the proposed method is the provision of estimates for separate components of the attributable risk, associated with different exposure ranges. In the specific case of temperature-health associations, this allows the separation of attributable risks from cold and heat, and further from mild and extreme temperatures. The estimates reported in the example highlights how the simple analysis of exposure-response curves can be misleading in the attribution of risk, and that most of the mortality in the two cities is in fact attributable to mild cold temperatures, in spite of the relatively low RR.

The availability of attributable risk measures, complementary to estimates of exposure-response associations, is essential for the identification and planning of public health interventions. Their extension to exposure-lag-response associations allows the computation of such measures from dependencies showing potentially complex non-linear and temporal patterns.

## Abbreviations

AF: Attributable fraction; AN: Attributable number of cases; RR: Relative risk; DLM: Distributed lag models; DLNM: Distributed lag non-linear models; eCI: empirical confidence interval.

## Competing interests

The authors declare that they have no competing interests.

## Authors’ contributions

AG conceived the idea of attributable risk measures for DLNMs and worked out their algebraic definitions. AG and ML developed the final version of the measures, planned the example and carried out the analysis, drafted the final version of the manuscript. AG provided the software implementation through the R scripts. Both authors read and approved the final manuscript.

## Pre-publication history

The pre-publication history for this paper can be accessed here:

http://www.biomedcentral.com/1471-2288/14/55/prepub

## Supplementary Material

Additional file 1 R script implementing the function to compute attributable risk measures. Click here for file

Additional file 2 Documentation of the attrdl function. Click here for file

Additional file 3 R script for fitting the regression models in the example. Click here for file

Additional file 4 R script for computing attributable risk in the example. Click here for file

Additional file 5 R script for producing the graphs in the example. Click here for file

Additional file 6 File with data used in the example. Click here for file
