# Supplementary material for: Attributable risk from distributed lag models
Source: BMC Med Res Methodol. 2014 Apr 23;14:55. doi: 10.1186/1471-2288-14-55 (PMC4021419; doi:10.1186/1471-2288-14-55)
Supplement: Additional file 2 — Documentation of the attrdl function. [file 1471-2288-14-55-S2.pdf]

R documentation

of ‘attrdl.Rd’

April 16, 2014

---

attrdl *Attributable Risk from a DLNM*

Description

This function computes estimates of attributable risk (fractions and numbers) from a distributed lag non-linear model (DLNM).

Usage

```
attrdl(x, basis, cases, model=NULL, coef=NULL, vcov=NULL, type="af", dir="back",
  tot=TRUE, range=NULL, sim=FALSE, nsim=5000)
```

Arguments

x	exposure vector or matrix of exposure histories for which the attributable risk needs to be computed.
basis	an object of class "crossbasis" used for fitting the model.
cases	the vector of cases or matrix of future cases corresponding to x.
model	the fitted model.
coef, vcov	user-provided coefficients and (co)variance matrix.
type	measure of attributable risk, either "af" (fraction, default) or "an" (number).
dir	direction used for computing the attributable risk, either "back" (backward, default) or "ford" (forward).
tot	logical. If TRUE (default), the total estimate is returned.
range	the range of exposure. If NULL, the whole range is used.
sim	logical. If TRUE, simulation samples are returned.
nsim	number of simulation samples (only for nsim=TRUE).

Details

This function computes the attributable fraction or number for a specific exposure scenario and associated cases, given an estimated exposure-lag-response association defined by a DLNM. Either *forward* or *backward* versions of attributable risk measures are available in thi setting. The method is described by Gasparrini and Leone (2014), see references below. The function works in combination with other functions in the package **dlnm**, which is assumed to be available.

The exposure and cases are provided by the arguments `x` and `cases`, respectively. The original cross-basis and fitted model containg it used for estimation are provided by the arguments `basis` and `model`, respectively. Alternatively, the user can provide estimated coefficients and (co)variance matrix with `coef` and `vcov`.

The function works both with time series and non-time series data. In a time series setting, both `x` and `cases` represent a complete series of ordered observations. More generally, the user can apply this function for any kind of data: in this case `x` must be a matrix of lagged exposures when `dir="back"`, and `cases` must be a matrix of future cases `dir="forw"`. The function can compute the total attributable risk (the default) or the contribution for each observation.

If `sim=TRUE`, the function computes samples of the attributable risk measures by simulating from the assumed normal distribution of the estimated coefficients (only implemented for total estimates). These samples can be used to defined empirical confidence intervals.

Value

By default, a numeric scalar or of total attributable fraction or number. If `sim=TRUE`, a vector of the simulated samples with length `nsim`. If `tot=FALSE`, a vector with contributions for all the observations.

Warnings

The code composing this function has not been systematically tested. The presence of bugs cannot be ruled out. Also, although written generically for working in different scenarios and data, the function has not been tested in contexts different than the example included in the article from Gasparrini and Leone (2014). It is responsibility of the user to check the reliability of the results in different applications.

Note

The functions can be also used with estimates from DLNMs reduced to the overall cumulative exposure-response through the function `crossreduce` in the package **dlnm**. In this case, the modified coefficients and (co)variance matrix of the reduced cross-basis in `basis` must be passed using the arguments `coef` and `vcov`. This option can be useful when the original estimates from the full cross-basis are not available any more, for example following a meta-analysis. Given the lag-specific estimates are not available in this case, only the forward version of attributable risk (`dir="forw"`) can be computed. See Gasparrini and Leone (2014) for further info.

Author(s)

Antonio Gasparrini, <antonio.gasparrini@lshtm.ac.uk>

References

Gasparrini A, Leone M. Attributable risk from distributed lag models. *BMC Medical Research Methodology*. 2014; [freely available here]

See Also

Functions in the package **dlnm**. See here for an updated version of this function.

Examples

```
# load the package dlnm and the function attrdl
library(dlnm)
source("attrdl.R")

# define the cross-basis and fit the model
cb <- crossbasis(chicagoNMMAPS$temp, lag=30, argvar=list(fun="bs",
  knots=c(-10,3,18),cen=21), arglag=list(knots=c(1,3,10)))
library(splines)
model <- glm(death ~  cb + ns(time, 7*14) + dow,
  family=quasipoisson(), chicagoNMMAPS)

# global backward attributable risk of temperature (number and fraction)
attrdl(chicagoNMMAPS$temp,cb,chicagoNMMAPS$death,model,type="an")
attrdl(chicagoNMMAPS$temp,cb,chicagoNMMAPS$death,model)

# global forward attributable fraction
attrdl(chicagoNMMAPS$temp,cb,chicagoNMMAPS$death,model,dir="forw")

# empirical confidence intervals
afsim <- attrdl(chicagoNMMAPS$temp,cb,chicagoNMMAPS$death,model,
  sim=TRUE,nsim=1000)
quantile(afsim,c(2.5,97.5)/100)

# attributable fraction component due to heat and cold
attrdl(chicagoNMMAPS$temp,cb,chicagoNMMAPS$death,model,range=c(21,100))
attrdl(chicagoNMMAPS$temp,cb,chicagoNMMAPS$death,model,range=c(-100,21))

# daily attributable deaths in the second month
attrdl(chicagoNMMAPS$temp,cb,chicagoNMMAPS$death,model,type="an",
  tot=FALSE)[31:60]
```

Index

attrdl, 1
